# The Application of Knowledge-Based Clinical Decision Support Systems to Detect Antibiotic Allergy

**DOI:** 10.3390/antibiotics13030244

**Published:** 2024-03-07

**Authors:** Nayoung Han, Ock Hee Oh, John Oh, Yoomi Kim, Younghee Lee, Won Chul Cha, Yun Mi Yu

**Affiliations:** 1 Jeju Research Institute of Pharmaceutical Sciences, College of Pharmacy, Jeju National University, Jeju 63243, Republic of Korea; 2 FirstDIS Ltd., Seoul 07343, Republic of Korea; ohoh@firstdis.co.kr; 3 Kakao Healthcare Corp., Seongnam 13529, Republic of Korea; john.csoh@kakaohealthcare.com; 4 Korea Health Information Service, Seoul 04512, Republic of Korea; ymkim@k-his.or.kr; 5 Department of Pharmacy, Ajou University Hospital, Suwon 16499, Republic of Korea; sidmom@hanmail.net; 6 Department of Digital Health, Samsung Advanced Institute for Health Science & Technology (SAIHST), Sungkyunkwan University, Seoul 06355, Republic of Korea; wc.cha@samsung.com; 7 Department of Pharmacy and Yonsei Institute of Pharmaceutical Sciences, College of Pharmacy, Yonsei University, Incheon 21983, Republic of Korea

**Keywords:** clinical decision support systems, antibiotics, drug allergy, knowledge database

## Abstract

Prevention of drug allergies is important for patient safety. The objective of this study was to evaluate the outcomes of antibiotic allergy-checking clinical decision support system (CDSS), K-CDS^TM^. A retrospective chart review study was performed in 29 hospitals and antibiotic allergy alerts data were collected from May to August 2022. A total of 15,535 allergy alert cases from 1586 patients were reviewed. The most frequently prescribed antibiotics were cephalosporins (48.5%), and there were more alerts of potential cross-reactivity between beta-lactam antibiotics than between antibiotics with the same ingredients or of the same class. Regarding allergy symptoms, dermatological disorders were the most common (38.8%), followed by gastrointestinal disorders (28.4%). The 714 cases (4.5%) of immune system disorders included 222 cases of anaphylaxis and 61 cases of severe cutaneous adverse reactions. Alerts for severe symptoms were reported in 6.4% of all cases. This study confirmed that K-CDS can effectively detect antibiotic allergies and prevent the prescription of potentially allergy-causing antibiotics among patients with a history of antibiotic allergies. If K-CDS is expanded to medical institutions nationwide in the future, it can prevent an increase in allergy recurrence related to drug prescriptions through cloud-based allergy detection CDSSs.

## 1. Introduction

Adverse drug reaction (ADR) is defined as a harmful and unintended response to a drug that occurs at normal doses or is tested for the diagnosis. World Health Organization (WHO) classifies ADR into two types, type A (intrinsic) and type B (idiosyncratic) reactions depending on whether they are predictable [1]. Type A reactions refer to predictable reactions such as drug overdose and pharmacological reactions. In contrast, type B reaction is a drug-dose-independent, unpredictable, and ultimately harmful reaction, which occurs even at doses commonly used. Despite the appropriate use of medicines, type B ADRs may occur, which can cause problems directly related to patient safety. Type B reactions can be more fatal in that they are unpredictable and affect any organs including skin, kidney, liver, and blood cells. Type B reactions account for up to 10% of ADRs, and approximately 5–10% of patients experience type B reactions according to a survey on the incidence of ADRs [2]. Those reactions are a significant cause of extended length of hospitalization, morbidity, and mortality for patients; they also markedly increase the economic burden of patients due to the prolongation of hospital stay and additional treatments [3]. According to a study conducted in Korea, the length of hospitalization was extended by approximately 5 days in patients with ADRs, and the overall average medical cost was significantly more than two times higher in patients with ADRs compared to patients without ADRs [4].

Most of these unpredictable type B reactions are drug allergies, including drug hypersensitivity. Common drugs that cause drug allergy include anticonvulsant drugs, antipsychotics, and antibiotics. Drug-induced hypersensitivity reactions (DHRs) manifest as various reactions that can be immunologically mediated or non-immunologically mediated: type I, immediate in onset and mediated by immunoglobulin E (IgE) and mast cells/basophils; type II, delayed in onset and caused by immunoglobulin G (IgG)-mediated cell destruction; type III, delayed in onset and caused by immune complex deposition and complement activation; and type IV, delayed in onset and T cell-mediated. DHR is difficult to detect using standard testing methods because the pattern and severity of clinical appearance vary from patient to patient [5,6]. As a result, predicting and preventing the occurrence of allergies in advance is the best strategy; however, the primary prevention of drug allergies has limitations owing to their diverse pathophysiology and causes [7,8]. Patients with a history of drug allergies are more likely to experience allergic reactions caused by other drugs with structurally and chemically similar properties through the involvement of T cells [9]. Moreover, repeated exposure to allergy-inducing drugs can lead to fatal consequences [10]. Therefore, secondary prevention, which is more feasible than primary prevention, is crucial in patients with a history of drug allergies.

Clinical decision support systems (CDSSs) that provide targeted clinical knowledge and patient-specific information [11] have the potential to prevent the occurrence of secondary drug allergies. CDSSs review the possibility of allergy incidence using information collected from patients and provide an alert when there is a high possibility of allergy caused by similar drugs [12]. Several studies have shown the positive effects of drug allergy prevention systems, including improvement of practitioner performance in drug selection and dose optimization [13,14,15]. Evans et al. showed that ADRs caused by antibiotics were reduced by more than 80% after implementation of CDSSs, and Heard et al. reported that inappropriate use of antibiotics decreased by 49% due to CDSSs. The importance of drug allergy prevention using CDSSs has also become increasingly important in terms of precision medicine and the reduction in unnecessary medical costs [14]. However, most current in-house CDSSs are hard-coded into electronic health records (EHR) in a single institution. Because the knowledge database is not easily shareable [16], health information must be accumulated individually by each institution. Therefore, these CDSSs are limited in that they are not checked when the patient visits another institution where there is no information about the history of drug allergies [17]. In particular, when an unconscious patient is brought to a hospital, serious problems can occur because proper decision-making to avoid allergy-causing drugs is challenging. However, there is still insufficient evidence on the incidence of cases of secondary allergy cases missed by the CDSS due to the lack of an information-sharing system.

**Figure 1:**
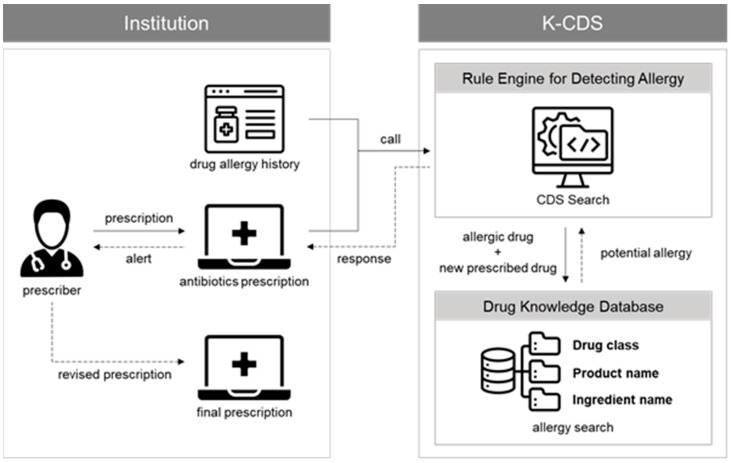
Cloud-based clinical decision support system (K-CDS^TM^) for checking drug allergy. CDS, clinical decision support.

To solve these problems, various cloud-based information-sharing systems have been developed with the benefit of ubiquitous access to relevant and timely personal health record data. The Korea Health Information Service (KHIS) developed K-CDS^TM^, a cloud-based CDSS to prevent the use of allergy-causing drugs through medical information sharing, built on the Fast Healthcare Interoperability Resource (FHIR)-based HL7 (the Health Level 7 standards organization) Clinical Decision Support (CDS) Hooks technical standard [18]. CDSSs are frequently classified as knowledge-based or non-knowledge based. In knowledge-based CDSSs, rules are created by using literature-based or practice-based evidence, and CDSS returns the results of a combination of a knowledge base and a reasoning engine rule to the user [19]. K-CDS is a system that can check drug allergies in real time by transmitting drug prescription information and the patient’s allergy history information, and it can send the results on the risk of drug allergy using the knowledge database to clinicians (see Figure 1). CDS services can be used simultaneously by multiple medical institutions with their own EHR systems. When a healthcare provider prescribes medication, the EHR system uses a remote CDS service call to transmit the patient’s prescription and allergy information, and K-CDS tests the risk of allergies and returns the results to the EHR system. Therefore, healthcare providers can check the possibility of drug allergies in real time.

In 2021, K-CDS included an interactive detection program for antibiotic allergies to confirm the possibility of allergy by linking a relational knowledge database on allergic cross-reactivity that may occur between drugs that belong to the same class or have similar structures or the same ingredients as the allergy-causing antibiotics [20]. The purpose of this study was to measure the characteristics of antibiotic allergy alerts using K-CDS and to evaluate the usefulness of K-CDS in allergy prevention.

## 2. Results

### 2.1. Results of Drug Allergy Checks Using K-CDS

**Figure 2:**
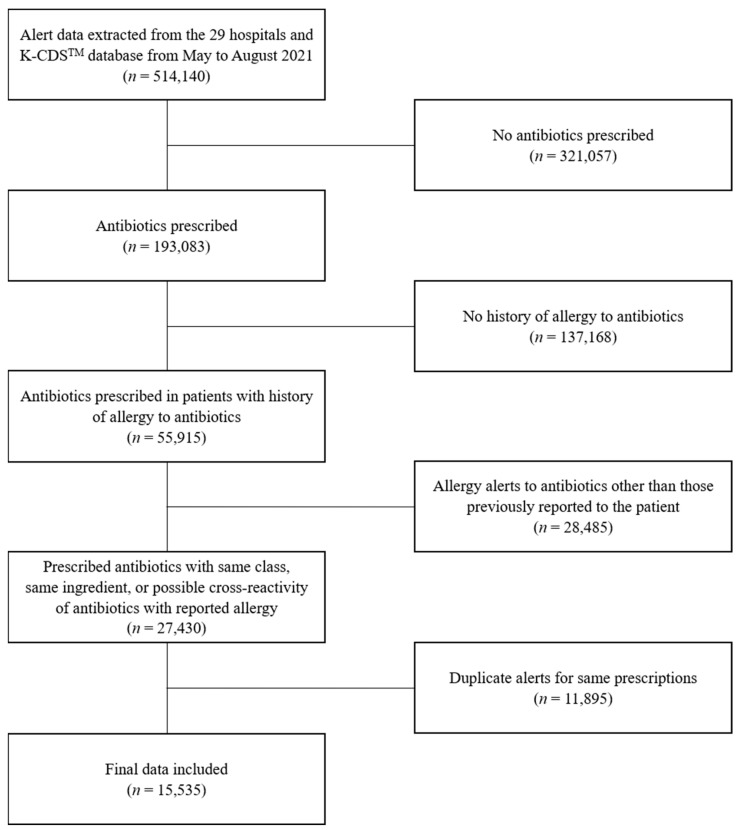
Flow diagram of data collection.

From a total of 514,140 prescription data, 321,057 prescriptions not including antibiotics were excluded. Afterwards, 137,168 prescriptions from patients who were not allergic to antibiotics were excluded. Then, 28,485 cases in which antibiotics from a class other than the antibiotic to which the patient exhibited an allergy were prescribed and drug allergy alerts that overlapped more than twice for the same prescription were excluded. Finally, 15,535 allergy alert cases from 1586 patients were selected as valid allergy check cases suitable for analysis based on the inclusion and exclusion criteria (Figure 2).

Men accounted for 45.3% of the patients. Adults between 19 and 65 years of age comprised the largest portion of patients (57.7%), followed by older adults over 65 years of age (36.9%) and children under 19 years of age (5.4%) (Table 1). Within the 29 medical institutions, 10,186 (65.6%) cases were detected in three tertiary hospitals, 5033 (32.4%) in 22 general hospitals, and 316 (2.0%) in four hospitals. Allergy checks were most frequently conducted in inpatient care (85.3%) in hospital settings, with 55.7% in internal medicine departments. This was followed by over 100 antibiotic allergy checks per month performed in pediatrics (1083 cases), emergency medicine (828 cases), and obstetrics and gynecology (462 cases) departments.

### 2.2. Frequency of Antibiotic Allergy Checks

Regarding detection type, 42.2% of the allergy alerts were identified as cross-reaction, which occurs in cases of drugs with a structure closely similar to that of allergy-causing antibiotics, followed by drugs with the same classes (26.5%) and ingredients or products (27.9%) (Table 2). More than three-quarters of the antibiotics checked were beta-lactams (78.6%). The most frequently checked antibiotics included cephalosporins (48.5%), penicillins (21.6%), and carbapenems (8.5%).

### 2.3. Symptoms and Severity of Checked Antibiotic Allergies

Regarding allergic symptoms, dermatologic disorders such as rash, flares, and hives were the most common (6184 cases, 38.8%), followed by gastrointestinal disorders such as diarrhea, nausea, and vomiting (4526 cases, 28.4%) (Table 3). Immune system disorders (714 cases, 4.5%) included 222 cases of anaphylaxis and 61 cases of severe cutaneous adverse reactions (SCARs), including 14 cases of Stevens–Johnson syndrome (SJS) and 47 cases of drug reactions with eosinophilia and systemic symptoms (DRESS). The number of checked allergies for vascular disorders was 126 (0.8%). Although the number was not high, one-third (31.7%) of cases had severe allergic reactions, such as hypovolemic shock. Alerts for severe and mild or moderate symptoms were reported in 6.4% and 78.2% of the cases, respectively. However, there was no information on severity in 13.8% of the patients.

## 3. Discussion

This study was the first to analyze the results of detecting the risk of antibiotic allergies with a common rule engine-based knowledge database that collects information from each medical institution and checks for possible allergies in a cloud-based CDSS in Korea. Most of the antibiotics prescribed during the period were checked well using the Identification of Medicinal Products (IDMP) codes.

Most antibiotic allergies were checked in inpatient care at tertiary hospitals, and more than one-third were observed in older adults aged over 65 years. Moreover, severe ADRs were reported in 6.4% of the patients, accounting for approximately one-third of the immune system and vascular disorders related to antibiotic allergies. These results demonstrate that K-CDS, a cloud-based CDSS, could be a valuable system for preventing the recurrence of severe antibiotic allergy, especially in older patients. It is well known that preventable adverse events occur twice as often in older adults than in younger people during hospitalization [21]. Older patients have a high risk of ADRs or drug allergies owing to the high rate of complex medication use for chronic diseases [22]. The polypharmacy rate among older people is >80% [23], and medications such as sedatives, antidepressants, and antihypertensives may increase the risk of anaphylaxis [24]. In addition, the frequency of antibiotic use among older patients is high owing to treatment or prophylaxis for infection after surgery [25], which increases the risk of recurrence if there is a history of antibiotic allergy. Therefore, applying K-CDS is expected to improve the safe use of drugs in older patients by preventing allergies.

Allergy checks averaged 3000 per institution (average of 750 per month) in the tertiary hospitals, and more than half of the alerts occurred in the Department of Internal Medicine. High-risk, severely ill patients are admitted to tertiary teaching hospitals and use many drugs because of acute and complex diseases; therefore, there is a high risk of adverse reactions such as allergies caused by the drugs [26]. Antibiotic allergy checks were frequently performed in the inpatient setting. Hospitalized patients are treated with various drugs owing to an unstable clinical condition [27]; in particular, antibiotics are often used to manage or prevent infection [28]. The department that frequently uses these antibiotics is internal medicine, and due to the characteristics of internal medicine patients, there is a high probability that they have chronic diseases and are also taking other drugs. Certain drugs, such as nonsteroidal anti-inflammatory drugs (NSAIDs), opioids, and biologics, have the potential to produce cross-reactions with antibiotics. In addition, more than 800 antibiotic allergy checks were conducted in emergency departments; this is a clinically important setting because drug allergies need to be prevented in an emergency even if the patient is unconscious. Because information obtained from an unconscious patient is very limited [29], cloud-based CDSSs for drug allergy checks are very useful for preventing the recurrence of allergies using information recorded in other hospitals.

In our study, most of the allergies had mild symptoms; however, many cases, including anaphylaxis and SCARs, were identified. In U.S. and U.K. cohort studies, the incidence of anaphylaxis or SCARs among all drug allergies was relatively low at 50 cases per 100,000 person-years [30,31]. Similar to foreign studies, in a study published in Korea, anaphylaxis occurred in 32 cases per 100,000 person-years in 2014 [32]. Although the number of SCARs checked through K-CDS was not high among the total allergies, the alert rate (1.43%) of anaphylaxis detected through K-CDS was much higher than that reported in previous epidemiological studies. In addition, the detection of severe immune disorders through the K-CDS can be expected to have economic benefits as well as clinical preventive effects [33,34]. In South Korea, a relief system for ADRs, which provides various relief benefits to patients and their families who suffer from death, disability, and newly diagnosed diseases caused by drug allergies or adverse reactions, was launched in 2013. The total amount of compensation paid in 2020 was KRW 1.97 billion including KRW 1.08 billion (54.6%) for death, KRW 490 million (24.8%) for medical expenses, KRW 320 million (16.0%) for disability, and KRW 0.9 billion (4.6%) for funeral expenses [35]. Therefore, preventing the recurrence of life-threatening allergies by K-CDS could reduce the socioeconomic burden on the government through a decrease in the payment for the relief of injury from severe ADRs.

Another notable feature of K-CDS is the use of globally standardized drug codes. The knowledge database of K-CDS was developed using IDMP codes to check antibiotic allergies more accurately and efficiently. IDMP use has been recommended to help with the unique identification of pharmaceuticals by the International Organization for Standardization (ISO), thereby ensuring the safety of medications [36]. There are several codes that identify drugs; however, among them, IDMP codes enable reliable identification of drugs by confirming the globally standardized ingredient names themselves [37]. In addition, K-CDS was developed to connect knowledge information to detect cases where the chemical structure of drugs is similar, even though the ingredient names are different [18]. As a result, the risk of allergies to the same product, same ingredient, same class, and even a closely related structure was well-checked. In our data, allergies to beta-lactam antibiotics, such as penicillins, cephalosporins, and carbapenems, were identified most frequently, which is similar to the findings of a previous study [38]. There is a high risk of cross-reaction between beta-lactam antibiotics owing to their structural similarity [39]; however, ascertaining cross-reactivity with only the brand names of the drugs is challenging [40]. Detection of potential cross-reactivity between antibiotics is an important role of knowledge-based CDSS. The defined daily dose per 1000 inhabitants per day (DID) of antibiotics is the highest for cephalosporins and the combination of penicillin and β-lactamase inhibitors worldwide [41]. Therefore, our K-CDS based on IDMP codes is useful as a global pharmacovigilance database for antibiotic treatment.

This study had several limitations. First, this was a pilot study and did not include sufficient alerts and resources due to the initial implementation of K-CDS. South Korea is preparing to build a nationwide network infrastructure system to enable the checking of drug allergies in real time by sharing health information among medical institutions [42]. In this respect, this pilot study helped evaluate the quality and performance of K-CDS and identify the challenges and limitations of expansions. Second, the clinical or economic outcomes were not evaluated. Previous studies showed that the occurrence of antibiotic allergies prolongs the length of hospitalization, resulting in additional medical costs [43,44]. Another potential limitation is that the secondary allergy prevention rate could not be calculated after applying K-CDS, because there was no allergy detection system before applying K-CDS. Therefore, further studies with complete clinical information are needed to confirm the outcomes of antibiotic allergy checks with the expansion of the system to more institutions in the future.

Furthermore, despite the effectiveness of K-CDS, its usefulness may be limited in clinical settings, which is thought to be because physicians ignore (alert override) the alerts about drug allergies provided by CDSS. In a study conducted in Korea, approximately 63.77% of prescription alerts for all medications in the emergency department were overridden, and the alert overridden rate of antibiotics was 67.83% [45]. As a result of analyzing characteristics related to alert override based on 2.7 million CDSS alert data from inpatient, outpatient, and emergency departments of a general hospital, the overridden rate (alert override count divided by alert count) was higher for the Department of Internal Medicine, physicians with more alerts, and most frequently prescribed drugs [46]. In other studies, the overridden rate ranged from 43.7% to 97%, and the main reason for override was that the allergic reaction was not fatal and that the patient previously tolerated the drugs without allergic reactions when re-administered [47]. In our study, we were unable to analyze data on prescription changes before and after the application of K-CDS, but the override rate is expected to be high because most of the alerts were mild-to-moderate symptoms. As allergy alerts to antibiotics increase, the fatigue of physicians inevitably increases, and alert override can lead to unanticipated outcomes such as an increased number of medication errors, resulting in threats to patient safety [48]. Therefore, in order for K-CDS to be applied more effectively clinically, future efforts will be important to reduce the override rate by optimizing the alert system providing clear information with the rationale of the alert and prioritizing alerts based on severity.

## 4. Materials and Methods

### 4.1. Data Collection

Data were collected to check for antibiotic allergies from 29 medical institutions in 1 special city, 4 metropolitan cities, and 5 provinces nationwide over 4 months from May to August 2021. Information corresponding to the patient’s medical history, diagnosis, and time of prescription was collected from the EHRs of each medical institution, and information corresponding to allergy checks was collected from the K-CDS cloud system. Drug allergy check data in K-CDS and EHR data from each medical institution were provided from the KHIS which has access to all information in the K-CDS database. All information was collected as anonymized data. Baseline information, including sex, age, type of medical institution, treatment environment, and department information, was extracted to analyze the characteristics of the patients. Only the antibiotic prescription data for patients with a history of antibiotic allergies were included during the medication period. Duplicate checks for the same drug for the same patient within 1 h were excluded. However, if the prescription date and allergy check time were different, the prescription was considered to be a different case. If two or more allergy histories were registered for one drug, or if multiple antibiotics were prescribed and checked, each check was considered a different case.

### 4.2. Antibiotic Allergy Check Analysis

Demographic characteristics were analyzed for cases in which antibiotic allergies were checked using K-CDS and analyzed by type of institution, environment, and medical department. Allergy check antibiotics were classified by ingredients and classes according to the IDMP code registered in the knowledge database of K-CDS. Drug allergic reactions were classified according to system organ class (SOC) based on the Medical Dictionary for Regulatory Activities (MedDRA) [49]. The severity of allergic reactions ranged from mild to life-threatening [50], as determined by the medical staff at the time of allergy registration.

### 4.3. Statistical Analysis

Data were analyzed using descriptive statistics and are presented as counts and percentages. All statistical analyses were performed using IBM SPSS statistics 24.0 (SPSS Inc., Chicago, IL, USA).

## 5. Conclusions

This pilot study showed that cloud-based drug allergy checks using K-CDS can be performed to prevent allergy recurrence among patients with a history of antibiotic allergies. If the K-CDS is expanded to other institutions and allergy information is shared more widely, positive outcomes in terms of medical cost reduction and safe drug use will be further enhanced, providing an opportunity to prevent the recurrence of drug allergies.

## Figures and Tables

**Table 1:** Descriptive characteristics of antibiotic allergy checks (*n* = 1586).

Variables		N	(%)
Gender ^1^	male	719	(45.3)
	female	867	(54.7)
Age ^1^	<19	86	(5.4)
	19 < 65	915	(57.7)
	≥65	585	(36.9)
Hospital level ^2^	tertiary	10,186	(65.6)
	secondary	5033	(32.4)
	primary	316	(2.0)
Setting ^2^	inpatients	13,245	(85.3)
	outpatients	1272	(8.2)
	emergency	936	(6.0)
	others	82	(0.5)
Department ^2^	internal medicine	8654	(55.7)
	orthopedics	1222	(7.9)
	general surgery	1157	(7.4)
	pediatrics	1083	(7.0)
	emergency	828	(5.3)
	neurology	541	(3.5)
	obstetrics and gynecology	462	(3.0)
	urology	295	(1.9)
	plastic surgery	258	(1.7)
	thoracic surgery	218	(1.4)
	otorhinolaryngology	218	(1.4)
	ophthalmology	161	(1.0)
	dentist	118	(0.8)
	anesthesiology and pain medicine	77	(0.5)
	rehabilitation medicine	71	(0.5)
	family medicine	42	(0.3)
	dermatology	30	(0.2)
	others	100	(0.6)

^1^ Number of patients; ^2^ Number of cases.

**Table 2:** Common allergy alert antibiotics (*n* = 15,535).

Category		*n* ^1^	(%)
Detection type	same ingredients	4123	(26.5)
	same class	4342	(27.9)
	cross-reactivity	6551	(42.2)
	detection error	519	(3.3)
Antibiotics class	cephalosporins	7539	(48.5)
	penicillins (including complex with beta-lactamase inhibitors)	3356	(21.6)
	carbapenems	1328	(8.5)
	glycoproteins	719	(4.6)
	quinolones	565	(3.6)
	sulfonamides	395	(2.5)
	aminoglycosides	72	(0.5)
	tetracyclines	25	(0.2)
	macrolides	9	(0.06)
	antibacterials—others	19	(0.1)
	nitroimidazoles	43	(0.3)
	antituberculosis	451	(2.9)
	antivirals	184	(1.2)
	antifungals—azoles	483	(3.1)
	antifungals—echinocandines	25	(0.2)
	antifungals—polyenes	322	(2.1)

^1^ Number of cases.

**Table 3:** Common allergic reactions and severity of antibiotic allergy alerts (*n* = 15,924).

Organ System Category	Mild	Moderate	Severe	Unknown	Total (%)
Dermatologic disorders	5667	31	213	273	6184 (38.8)
Gastrointestinal disorders	4297	48	119	62	4526 (28.4)
Immune system disorders	301	0	373	40	714 (4.5)
Nervous system disorders	550	0	70	19	639 (4.0)
General disorders and administration site conditions	513	4	85	1	603 (3.8)
Cardiac disorders	525	10	35	2	572 (3.6)
Investigations	235	5	49	135	424 (2.6)
Blood and lymphatic system disorders	141	0	33	0	174 (1.1)
Renal and urinary disorders	149	0	0	0	149 (0.9)
Vascular disorders	86	0	40	0	126 (0.8)
Vision disorders	57	0	0	0	57 (0.4)
Psychiatric disorders	41	0	0	0	41 (0.3)
Respiratory, thoracic, and mediastinal disorders	16	0	8	0	24 (0.2)
Metabolism and nutrition disorders	12	0	0	0	12 (0.1)
Musculoskeletal and connective tissue disorders	12	0	0	0	12 (0.1)
Ear and labyrinth disorders	2	0	0	0	2 (0.0)
Unknown	0	0	0	1665	1665 (10.4)

## Data Availability

Further inquiries about the data sets may be directed to the corresponding author.
